# Quality Measures Addressing Disparities to Improve Outcomes in Hand Surgery

**DOI:** 10.1177/15589447261453556

**Published:** 2026-06-23

**Authors:** Emily A. Schultz, Eli M. Snyder, Marc J. Richard, David S. Ruch, David Ring, Sanjeev Kakar, Christopher Got, Edward Akelman, Philip E. Blazar, Jeffrey Yao, Amy L. Ladd, Erika D. Sears, Robin N. Kamal, Lauren M. Shapiro

**Affiliations:** 1 Stanford University, Redwood City, CA, USA; 2 University of Hawaii, Honolulu, USA; 3 Duke University, Durham, NC, USA; 4 The University of Texas at Austin, USA; 5 Mayo Clinic, Rochester, MN, USA; 6 Brown University, Providence, RI, USA; 7 University Orthopedics, East Providence, RI, USA; 8 Brigham and Women’s Hospital, Boston, MA, USA; 9 University of Michigan, Ann Arbor, USA; 10 University of California, San Francisco, USA

**Keywords:** disparities, hand surgery, orthopedic surgery, quality measure, social determinants of health

## Abstract

**Background:**

There is growing evidence that social determinants of health (SDOH) are associated with disparities in access to care and hand and upper extremity health. Quality measures represent an opportunity to evaluate disparities in access and outcomes that can inform improvement interventions. We performed a systematic review of evidence regarding health disparities within hand surgery and aimed to develop suitable quality measures that are clinically important, feasible, usable, and scientifically acceptable.

**Methods:**

We performed a systematic review including common hand surgery terms to identify health disparities in hand surgery related to SDOH. Candidate quality measures were constructed based upon evidence from the systematic review. A consortium of 11 US-based hand and upper extremity surgeons completed a modified RAND/UCLA Delphi Appropriateness process to evaluate the importance, feasibility, usability, and scientific acceptability of the candidate quality measures. Panelists rated each measure on a scale of 1 (*definitely not important/feasible/usable/scientifically acceptable*) to 9 (*definitely important/feasible/usable/scientifically acceptable*) in 2 voting rounds separated by a face-to-face discussion. Agreement among panelists and validity were assessed using predetermined criteria.

**Results:**

Fourteen candidate quality measures addressing health disparities were identified based on evidence from the systematic review, including time to surgery and emergency department use after hand surgery based on insurance type, clinical outcomes based on social deprivation, among others. All 14 measures were accepted.

**Conclusions:**

Fourteen candidate quality measures were identified and accepted based upon consensus to address health disparities in hand surgery, although future investigation will be needed to evaluate their effectiveness.

## Introduction

The management and outcomes of hand and upper extremity conditions may be affected by social determinants of health (SDOH), which are defined as the conditions in the environments where people are born, live, learn, work, play, worship, and age that affect a wide range of health, functioning, and quality-of-life outcomes and risks.^1^ Social determinants of health are estimated to account for 30% to 55% of an individual’s health outcomes and contribute to health disparities related to unequal access to care.^2^ In orthopedic surgery, orthopedic trauma patients with educational deficiencies and lower economic status had increased rates of readmission, revision surgery, and major complications following hip and ankle fracture surgery.^3^ In hand surgery, increases in the number of adverse SDOH were associated with increased complications following distal radius fracture fixation.^4^ Another study found patients who were uninsured or who had Medicaid insurance were significantly less likely to complete follow-up care after acute soft-tissue or bony injuries of the hand or wrist.^5^ Similarly, health-related social needs (HRSNs), which are more individualized patient-reported drivers of health, affect an individual’s ability to maintain their health and are often the downstream effects of SDOH.^6^ Given the role of HRSNs in patient care, the Centers for Medicare & Medicaid Services (CMS) had initially instituted policies for the screening of social drivers of health, however this was not included in the final rule in 2026. However, there remains a paucity of tools which measure SDOH among patients to inform improvement interventions, particularly in orthopedic surgery.^7,8^

Quality measures are tools that quantify health care processes, outcomes, and organizational structures. Data from quality measures may be used to assess institution or clinician performance and to evaluate quality improvement interventions.^9^ As a result, quality measures can be used as a lever to improve health care quality and patient outcomes.^10^ In orthopedic surgery, public reporting of quality measures has been associated with reduced complication and readmission rates in patients undergoing total joint arthroplasty.^11^ In 1 meta-analysis across surgical fields, public reporting was associated with a reduction in mortality rates.^12^ While previous studies emphasize the effectiveness of the public reporting of existing quality measures, there remains a gap in the development of equity-based quality measures that may address health disparities identified in current studies, such as poorer outcomes for patients based on insurance status. Quality measures focused on improving health equity may be used to identify institutional opportunities for quality improvement based on existing health disparities.^13^

We performed a systematic review to identify quality measures evaluating health disparities as measured by SDOH within hand surgery.

## Methods

### Literature Review

A systematic review was conducted to identify health disparities as measured by SDOH in hand surgery, as defined by Healthy People 2030, which includes economic stability, education access and quality, health care access and quality, neighborhood and built environment, and social and community context.^1^ Healthy People 2030 is a globally recognized initiative from the U.S. Department of Health and Human Services that includes data-driven, measurable objectives with 10-year targets to improve health.^14^ The objectives set by Healthy People 2030 have been established since 1979 and remain one of the most common measures of achieving health equity.^15^ This systematic review followed the Preferred Reporting Items for Systematic Reviews and Meta-Analyses (PRISMA) guidelines.^16^ The MEDLINE/PubMed medical database was used. We created customized search criteria with common hand surgery and SDOH terms from prior literature and the PROGRESS framework such as “hand,” “wrist,” “elbow,” and “surgery” as well as “place of residence,” “insurance,” “socioeconomic,” “occupation,” “employment status,” “race,” “ethnicity,” “religion,” “education,” “social capital,” “social determinants,” “gender,” or “sex” to reflect the SDOH defined by Healthy People 2030 (Supplemental Appendix Table A1 and Table A2).^17-19^ The MEDLINE/PubMed search was performed on July 7, 2024.

Articles discussing health disparities in hand surgery related to SDOH were included if they described conditions affecting the hand, wrist, elbow, or finger, and if they were full-length, US-based studies published in English. Exclusion criteria comprised articles without full-length text, non-US-based studies, systematic reviews, publication date before 2014, and studies related to conditions affecting the shoulder. Articles identified from the MEDLINE/PubMed search were uploaded to Covidence to facilitate the initial screening of article titles and abstracts. Titles and abstracts were independently reviewed by 2 authors (EMS and EAS), and any conflicts were resolved via consensus among 2 senior authors (RNK and LMS). The same 2 authors who reviewed titles and abstracts reviewed the full text of the manuscripts that passed the initial screening, and any conflicts were resolved via consensus among 2 senior authors. Additional papers were included if they were identified among references from literature captured by the MEDLINE/PubMed search. Articles were classified by the SDOH domains defined by Healthy People 2030. One specific metric included in the systematic review was social deprivation. Social deprivation is measured by the Area Deprivation Index (ADI) and is a validated zip-code measure that accounts for factors such as income, education, and housing quality. ADI has been identified as a proxy for socioeconomic status in outpatient orthopedic surgery patients. Worse ADI is correlated with worse function, complications, and outcomes.^20,21^

### Quality Measure Development

Evidence from the systematic review was used to develop candidate quality measures. Candidate quality measures were drafted and finalized by 2 senior authors (RNK and LMS). The evidence and candidate measures were sent to the members of the Hand Surgery Quality Consortium (HSQC). The HSQC is comprised of fellowship-trained hand and upper extremity surgeons and/or experts in quality measure development. HSQC members were invited to submit additional topics or measures that they felt were relevant. Candidate measures and their supporting evidence were compiled and evaluated by an 11-person panel of HSQC members through a modified RAND/UCLA (University of California Los Angeles) Delphi Appropriateness process to evaluate the importance, feasibility, usability, and scientific acceptability of each candidate measure.^22^ The Delphi process combines the highest level of available evidence with expert judgment, and it is used when high-level evidence does not exist or is not applicable.^23^ Thus, the Delphi Appropriateness methodology produces appropriateness criteria and quality measures based on group consensus.^23-26^

### RAND/UCLA Delphi Scoring

The Delphi voting process included 2 rounds of independent ratings of the quality measures (preliminary and final), with a face-to-face group discussion between the 2 rounds of voting. Each measure was rated according to the 4 criteria (importance, feasibility, usability, and scientific acceptability) on a scale of 1 to 9, where 1 was *definitely not important/feasible/usable/scientifically* acceptable, 5 *was uncertain or equivocal importance/feasibility/usability/scientific acceptability*, and 9 was *definitely important/feasible/usable/scientifically acceptable* in their practice setting. Panelists were provided scoring instructions prior to voting. According to the Delphi methodology, measures reached consensus if they received a median score of 7 or higher for all 4 criteria with no more than 3 panelists rating outside of the 7 to 9 range for all criteria.^23^ All members of the HSQC have prior experience completing the RAND/UCLA Delphi methodology.

### Voting Rounds

During the voting process, panelists were prompted to provide open-ended responses/comments to discuss at the in-person meeting. Panelists were provided with the aggregate scores at the meeting, and the HSQC chair (RNK) facilitated a discussion regarding the importance, feasibility, usability, and scientific acceptability of the candidate measures. The candidate measures were revised based on the discussion at the meeting, and the updated measures were shared with the panelists to complete a second round of voting within 2 weeks of the meeting.

The chair of the HSQC (RNK) facilitated the consortium discussion during the US-based face-to-face meeting, but did not participate in voting. All HSQC members are hand surgeons with previous experience in quality measure development and with diverse practice patterns in a variety of geographical locations.

## Results

**Figure 1.**
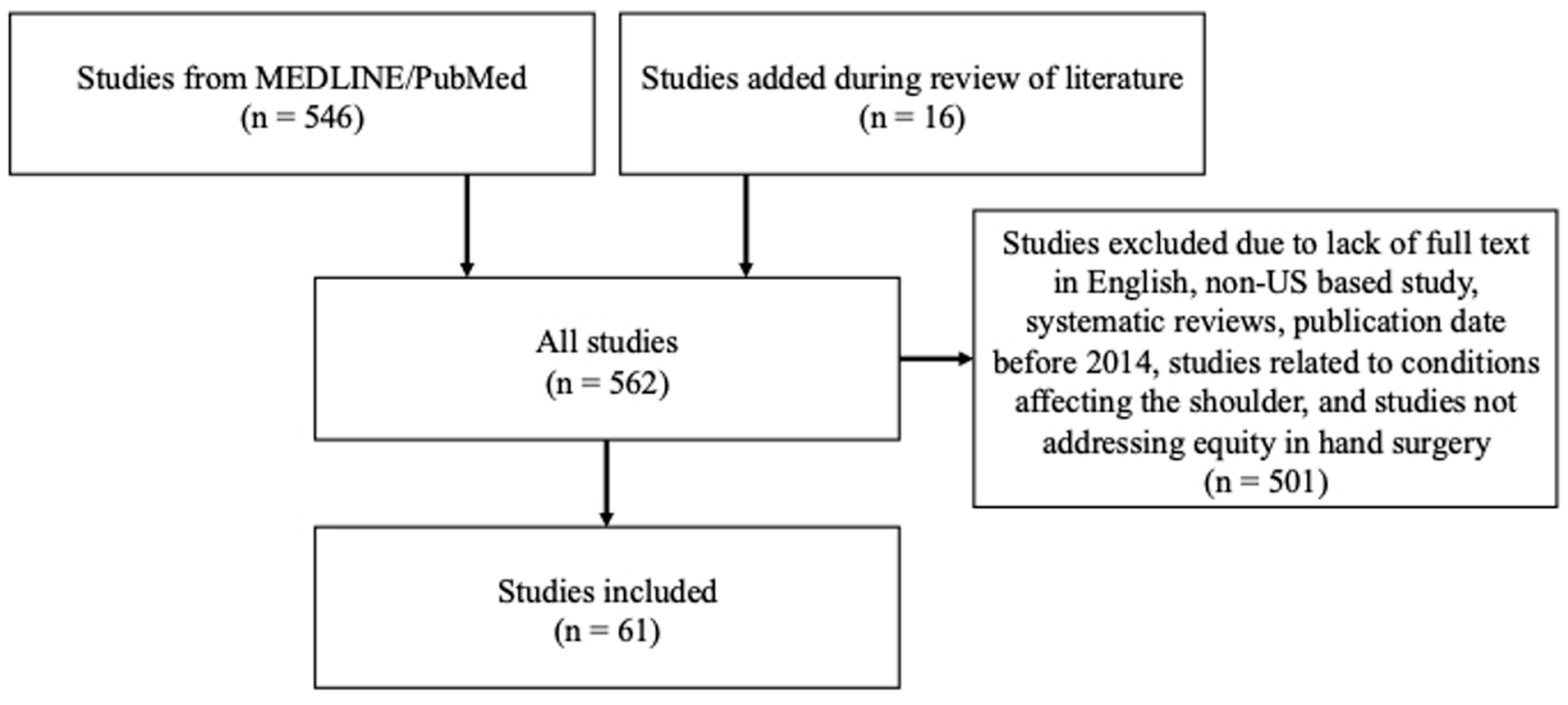
The Preferred Reporting Items for Systematic Reviews and Meta-Analyses (PRIMSA) flowchart for studies that were identified and selected for the systematic review.

The initial MEDLINE/PubMed search yielded 546 articles. During the review of the literature, 16 additional papers not captured by the MEDLINE/PubMed search were included following citation review. After review of all articles, 501 were excluded (Figure 1). In total, 61 articles relevant to health disparities in hand surgery were identified. Several articles addressed more than 1 of the 5 defined SDOH domains, with defined domains addressed a total of 79 times within the 61 included articles. Health care access and quality (n = 27, 44%) and social and community context (n = 24, 39%) were the domains most frequently discussed, whereas neighborhood and built environment (n = 1, 2%) was the least investigated domain (Table 1).

**Table 1.** SDOH Domains Addressed in Literature Related to SDOH in Hand Surgery.

SDOH domain	Domain count (%)
Health Care Access and Quality	27 (44)
Social and Community Context	24 (39)
Education Access and Quality	17 (29)
Economic Stability	10 (16)
Neighborhood and Built Environment	1 (2)
Total (among 61 articles)	79

Abbreviation: SDOH, social determinants of health.

Fourteen candidate quality measures addressing health disparities in hand surgery were chosen based on evidence from the systematic review (Table 2). All 14 candidate measures received a median rating of 9 for importance, feasibility, usability, and scientific acceptability. Therefore, all candidate measures were considered valid based on agreement among the consortium’s ratings across the 4 voting domains. These measures (Table 2) addressed health disparities related to time to surgery and emergency department use after hand and upper extremity surgery based on insurance type, clinical outcomes based on social deprivation, postoperative patient reported outcom measure (PROM) completion rate based on social deprivation and race, and mean morphine milligram equivalent of postoperative opioid prescriptions and length of hospital stay based on race.

**Table 2.** Final Candidate Quality Measures Proposed Based on Evidence From the Systematic Review, With Revisions From the HSCQ in-Person Meeting Incorporated.

#	Quality concept	Implementation level	Measure type	Measure specification	Numerator	Denominator
1	There should be no difference in time to surgery for patients with carpal tunnel syndrome based on insurance type.	Group practice Tax ID Number (TIN) level	Outcome	Ratio of the median number of days from diagnosis to surgery for patients with carpal tunnel syndrome with Medicare and/or Medicaid insurance vs those with commercial insurance.	Median time from diagnosis to surgery for patients undergoing carpal tunnel release with Medicare and/or Medicaid insurance	Median time from diagnosis to surgery for patients undergoing carpal tunnel release with commercial insurance
2	There should be no difference in time to surgery for patients with distal radius fractures based on insurance type.	Group practice (TIN) level	Outcome	Ratio of the median number of days from diagnosis to surgery for patients who received surgical treatment for distal radius fractures with Medicare and/or Medicaid insurance vs those with commercial insurance.	Median time from diagnosis to surgery for patients who received surgical treatment for distal radius fracture with Medicare and/or Medicaid insurance	Median time from diagnosis to surgery for patients who received surgical treatment for distal radius fracture with commercial insurance
3	There should be no difference in time to surgery for patients with a trigger finger based on insurance type.	Group practice (TIN) level	Outcome	Ratio of the median number of days from diagnosis to surgery for patients with trigger finger with Medicare and/or Medicaid insurance vs those with commercial insurance.	Median time from diagnosis to surgery for patients undergoing trigger finger release with Medicare and/or Medicaid insurance	Median time from diagnosis to surgery for patients undergoing trigger finger release for patients with commercial insurance
4	There should be no difference in time to surgery for patients with cubital tunnel syndrome based on insurance type.	Group practice (TIN) level	Outcome	Ratio of the median number of days from diagnosis to surgery for patients with cubital tunnel syndrome with Medicare and/or Medicaid insurance vs those with commercial insurance.	Median time from diagnosis to surgery for patients undergoing cubital tunnel release with Medicare and/or Medicaid insurance	Median time from diagnosis to surgery for patients undergoing cubital tunnel release for patients with commercial insurance
5	There should be no difference in time to surgery for patients with thumb carpometacarpal arthritis based on insurance type.	Group practice (TIN) level	Outcome	Ratio of the median number of days from diagnosis to surgery for patients with thumb carpometacarpal arthritis with Medicare and/or Medicaid insurance compared with those with commercial insurance.	Median time from diagnosis to surgery for patients undergoing thumb carpometacarpal arthroplasty with Medicare and/or Medicaid insurance	Median time from diagnosis to surgery for patients undergoing thumb carpometacarpal arthroplasty for patients with commercial insurance
6	There should be no difference in clinical outcomes after carpal tunnel release based on social deprivation.	Group practice (TIN) level	Outcome	Ratio of the percentages of patients undergoing carpal tunnel release surgery from the top 2 quintiles of zone improvement plan (ZIP)-code based social deprivation (ADI) reaching a minimally clinically important differencen (MCID) of 10 points^41^ on the Quick Disabilities of the Arm, Shoulder, and Hand (QuickDASH) compared with those from the bottom 2 quintiles of ZIP-code based social deprivation scores.	Percentage of patients reaching an MCID of 10 points on the QuickDASH within 1 year after carpal tunnel release from the top 2 quintiles of ZIP-code based social deprivation scores.	Percentage of patients reaching an MCID of 10 points on the QuickDASH within 1 year after carpal tunnel release from the bottom 2 quintiles of ZIP-code based social deprivation scores.
7	There should be no difference in clinical outcomes after surgical treatment for distal radius fractures based on social deprivation.	Group practice (TIN) level	Outcome	Ratio of the percentages of patients undergoing surgery for distal radius fractures from the top 2 quintiles of ZIP-code based social deprivation (ADI) reaching an MCID of 10 points^41^ on the QuickDASH compared with those from the bottom 2 quintiles of ZIP-code based social deprivation scores.	Percentage of patients reaching an MCID of 10 points on the QuickDASH within 1 year after distal radius fracture surgery from the top 2 quintiles of ZIP-code based social deprivation scores.	Percentage of patients reaching an MCID of 10 points on the QuickDASH within 1 year after distal radius fracture surgery from the bottom 2 quintiles of ZIP-code based social deprivation scores.
8	There should be no difference in clinical outcomes after surgical treatment of carpal tunnel syndrome based on social deprivation.	Group practice (TIN) level	Outcome	Ratio of the percentages of patients undergoing surgical treatment of carpal tunnel syndrome from the top 2 quintiles of ZIP-code based social deprivation (ADI) reaching an MCID of 5 points^41^ on the PROMIS PF within 1 year after surgery compared with those from the bottom 2 quintiles of ZIP-code based social deprivation scores.	Percentage of patients reaching an MCID of 5 points on the PROMIS PF within 1 year after surgery for carpal tunnel syndrome from the top 2 quintiles of ZIP-code based social deprivation scores.	Percentage of patients reaching an MCID of 5 points on the PROMIS PF within 1 year after surgery for carpal tunnel syndrome from the bottom 2 quintiles of ZIP-code based social deprivation scores.
9	There should be no difference in emergency department use after hand and upper-extremity surgeries (with diagnoses including carpal tunnel syndrome, cubital tunnel syndrome, trigger finger, distal radius fracture, Dupuytren disease, de Quervain’s tenosynovitis, wrist ganglion cyst, carpometacarpal arthritis, and scaphoid nonunion) based on insurance type.	Group practice (TIN) level	Outcome	Ratio of the number of emergency department visits for patients undergoing hand and upper-extremity surgeries (with diagnoses including carpal tunnel syndrome, cubital tunnel syndrome, trigger finger, distal radius fracture, Dupuytren disease, de Quervain’s tenosynovitis, wrist ganglion cyst, carpometacarpal arthritis, and scaphoid nonunion) with Medicare and/or Medicaid insurance vs those with commercial insurance.	Number of emergency department visits for patients undergoing hand and upper-extremity surgeries (with diagnoses including carpal tunnel syndrome, cubital tunnel syndrome, trigger finger, distal radius fracture, Dupuytren disease, de Quervain’s tenosynovitis, wrist ganglion cyst, carpometacarpal arthritis, and scaphoid nonunion) with Medicare and/or Medicaid insurance.	Number of emergency department visits for patients undergoing hand and upper-extremity surgeries (with diagnoses including carpal tunnel syndrome, cubital tunnel syndrome, trigger finger, distal radius fracture, Dupuytren disease, de Quervain’s tenosynovitis, wrist ganglion cyst, carpometacarpal arthritis, and scaphoid nonunion) with commercial insurance.
10	There should be no difference in PROM completion rate after hand and upper-extremity surgeries (with diagnoses including carpal tunnel syndrome, cubital tunnel syndrome, trigger finger, distal radius fracture, Dupuytren disease, de Quervain’s tenosynovitis, wrist ganglion cyst, carpometacarpal arthritis, and scaphoid nonunion) based on social deprivation.	Physician level	Outcome	Ratio of the rate of QuickDASH completion at 1 year for patients undergoing hand and upper-extremity surgeries (with diagnoses including carpal tunnel syndrome, cubital tunnel syndrome, trigger finger, distal radius fracture, Dupuytren disease, de Quervain’s tenosynovitis, wrist ganglion cyst, carpometacarpal arthritis, and scaphoid nonunion) from the top 2 quintiles of ZIP-code based social deprivation scores compared with those from the bottom 2 quintiles of ZIP-code based social deprivation scores.	Rate of QuickDASH completion at 1 year after hand and upper-extremity surgeries (with diagnoses including carpal tunnel syndrome, cubital tunnel syndrome, trigger finger, distal radius fracture, Dupuytren disease, de Quervain’s tenosynovitis, wrist ganglion cyst, carpometacarpal arthritis, and scaphoid nonunion) of those patients from the top 2 quintiles of ZIP-code based social deprivation scores.	Rate of QuickDASH completion at 1 year after hand and upper-extremity surgeries (with diagnoses including carpal tunnel syndrome, cubital tunnel syndrome, trigger finger, distal radius fracture, Dupuytren disease, de Quervain’s tenosynovitis, wrist ganglion cyst, carpometacarpal arthritis, and scaphoid nonunion) of those patients from the bottom 2 quintiles of ZIP-code based social deprivation scores.
11	There should be no difference in PROM completion rate after hand and upper-extremity surgeries (with diagnoses including carpal tunnel syndrome, cubital tunnel syndrome, trigger finger, distal radius fracture, Dupuytren disease, de Quervain’s tenosynovitis, wrist ganglion cyst, carpometacarpal arthritis, and scaphoid nonunion) based on race.	Physician level	Outcome	Ratio of the rate of QuickDASH completion at 1 year for patients undergoing hand and upper-extremity surgeries (with diagnoses including carpal tunnel syndrome, cubital tunnel syndrome, trigger finger, distal radius fracture, Dupuytren disease, de Quervain’s tenosynovitis, wrist ganglion cyst, carpometacarpal arthritis, and scaphoid nonunion) among nonwhite patients compared with white patients.	Percentage of nonwhite patients who completed QuickDASH at 1 year after hand and upper-extremity surgeries (with diagnoses including carpal tunnel syndrome, cubital tunnel syndrome, trigger finger, distal radius fracture, Dupuytren disease, de Quervain’s tenosynovitis, wrist ganglion cyst, carpometacarpal arthritis, and scaphoid nonunion).	Percentage of white patients who completed QuickDASH at 1 year after hand and upper-extremity surgeries (with diagnoses including carpal tunnel syndrome, cubital tunnel syndrome, trigger finger, distal radius fracture, Dupuytren disease, de Quervain’s tenosynovitis, wrist ganglion cyst, carpometacarpal arthritis, and scaphoid nonunion).
12	There should be no difference in mean MME of opioid prescriptions filled after carpal tunnel release between Latinx and white patients.	Physician level	Structure	Ratio of the mean MME of opioid prescriptions filled by Latinx patients compared with white patients within 30 days after carpal tunnel release.	Mean MME of opioid prescriptions filled by Latinx patients within 30 days after carpal tunnel release surgery.	Mean MME of opioid prescriptions filled by white patients within 30 days after carpal tunnel release surgery.
13	There should be no difference in mean MME of opioid prescriptions filled after carpal tunnel release between black and white patients.	Physician level	Structure	Ratio of the mean MME of opioid prescriptions filled by black patients compared with white patients within 30 days after carpal tunnel release.	Mean MME of opioid prescriptions filled by black patients within 30 days after carpal tunnel release surgery.	Mean MME of opioid prescriptions filled by white patients within 30 days after carpal tunnel release surgery.
14	There should be no difference in length of hospital stay following total elbow arthroplasty based on race.	Group practice (TIN) level	Outcome	Ratio of the median hospital length of stay among black patients compared with white patients after total elbow arthroplasty.	Median hospital length of stay among black patients who receive total elbow arthroplasty.	Median hospital length of stay among white patients who receive total elbow arthroplasty.

Abbreviations: ADI, Area Deprivation Index; MME, morphine milligram equivalent; PROMIS, patient-reported outcomes measurement information system; PF, physical function.

## Discussion

Quality measures may enable the identification of health disparities in hand surgery and thus could be used as tools to inform interventions and improve care quality. In this study, 14 candidate quality measures were developed based on a systematic review of the literature pertaining to health disparities within hand surgery, and there was agreement among the panelists as to their importance, feasibility, usability, and scientific acceptability.

The quality measures developed in this study align with established measures that address health inequities in fields outside of orthopedic surgery. For example, in 2025, the National Committee for Quality Assurance (NCQA) included race and ethnicity in quality measure stratification for measures focused on access to care and care use.^27^ One NCQA quality measure, Prenatal and Postpartum Care, focuses on access and availability of care. Stratification of this quality measure by race and ethnicity allows systems to identify health disparities in access to necessary health care services. These measures are used not only to evaluate quality and performance, but have been integrated into payment models to promote value-based care.^28^ Using the developed quality measures to track performance and influence reimbursement models may be an initial step toward acknowledging and reducing health disparities. In addition, quality measures released by orthopedic surgery organizations and societies may include equity-based measures to track and incentivize reductions in care inequities. In this study, 2 quality measures focused on differences in outcomes for common hand surgery procedures between patients of different races. These quality measures may identify race-based differences in hand surgery identifying opportunities for improvement, which could be used to inform interventions (eg, development of care pathways) to mitigate disparities.

Accountable care organizations (ACOs) may benefit from SDOH assessment within quality measurement. ACOs are a group of physicians, health care professionals, and hospital systems that coordinate the delivery of efficient patient care, set benchmarks for quality, efficiency, and cost-effectiveness of care.^29^ The ACO Realizing Equity, Access, and Community Health (REACH) model aims to promote health equity by bringing accountable care to underserved communities.^30^ Equity-based quality measures may be implemented into ACOs to identify opportunities in reducing health inequities and improving care delivery. For example, within the ACO REACH model, ADI has been implemented to evaluate the delivery of care to patients in underserved populations.^31,32^ This metric accounts for health disparities based on geographic location in quality measurement. However, other variables, such as differences in race and ethnicity, may lead to the exacerbation of existing health disparities. For example, ACOs may be incentivized to include patients who are more likely to achieve favorable health outcomes and who do not face systemic barriers to care. Incorporating equity-based quality measures into ACO quality frameworks may ensure health inequities are effectively addressed rather than exacerbated.

This study has limitations. For example, there is a risk of conformity bias inherent to the Delphi methodology.^33,34^ To reduce potential bias, voting was conducted anonymously. In addition, all HSQC participants have prior experience in quality measure development and with the Delphi methodology, which may reduce the risk of bias and error. In addition, the HSQC panel is not representative of all hand surgeons and there is risk of selection bias. The HSQC panel is comprised of 11 hand surgeons, and their perspectives may not be representative of the perspectives and experiences of all hand surgeons in the United States. We aimed to include hand surgeons from a diverse array of practice patterns, geographic locations, and professional experiences to incorporate a range of expert opinions. These opinions may inevitably vary based on factors such as geographic location and professional experiences. However, measures underwent several revisions based on feedback from the HSQC, including an intervening face-to-face discussion, which may have reduced potential bias. Although an extensive literature review was completed, it is possible that not all studies capturing SDOH in hand surgery were included. This was mitigated through having 2 independent reviews of the included studies by 2 authors with reference searching to include relevant studies that may not have been initially identified. The studies included in the literature review are limited to US-based studies to ensure consistency and applicability, as members of the HSQC currently practice in the United States. However, future consensus work may include internationally-based hand surgeons to improve global generalizability. In addition, these quality measures have not been validated in practice, and there may be limitations to their clinical applicability.

Fourteen quality measures addressing health disparities in hand surgery were developed based on a systematic review of the literature and a modified RAND/UCLA Appropriateness process. These measures may help to identify health disparities in hand surgery and enable equity-driven incentives to decrease health disparities related to SDOH. Future research may investigate quality measure implementation strategies and assess the impact of incorporating equity-based quality measurement into clinical practice.

## Supplemental Material

sj-docx-1-han-10.1177_15589447261453556 – Supplemental material for Quality Measures Addressing Disparities to Improve Outcomes in Hand Surgery Supplemental material, sj-docx-1-han-10.1177_15589447261453556 for Quality Measures Addressing Disparities to Improve Outcomes in Hand Surgery by Emily A. Schultz, Eli M. Snyder, Marc J. Richard, David S. Ruch, David Ring, Sanjeev Kakar, Christopher Got, Edward Akelman, Philip E. Blazar, Jeffrey Yao, Amy L. Ladd, Erika D. Sears, Robin N. Kamal and Lauren M. Shapiro in HAND

sj-docx-2-han-10.1177_15589447261453556 – Supplemental material for Quality Measures Addressing Disparities to Improve Outcomes in Hand Surgery Supplemental material, sj-docx-2-han-10.1177_15589447261453556 for Quality Measures Addressing Disparities to Improve Outcomes in Hand Surgery by Emily A. Schultz, Eli M. Snyder, Marc J. Richard, David S. Ruch, David Ring, Sanjeev Kakar, Christopher Got, Edward Akelman, Philip E. Blazar, Jeffrey Yao, Amy L. Ladd, Erika D. Sears, Robin N. Kamal and Lauren M. Shapiro in HAND
