# Supplementary material for: Quality Measures Addressing Disparities to Improve Outcomes in Hand Surgery
Source: Hand (N Y). 2026 Jun 23:15589447261453556. Online ahead of print. doi: 10.1177/15589447261453556 (PMC13294116; doi:10.1177/15589447261453556)
Supplement: sj-docx-1-han-10.1177_15589447261453556 – Supplemental material for Quality Measures Addressing Disparities to Improve Outcomes in Hand Surgery [file sj-docx-1-han-10.1177_15589447261453556.docx]

**Appendix**

**Table A1**: Search terms relevant to hand surgery and social determinants of health (SDOH), as well as the complete MEDLINE/PubMed query.

| **Relevance** | **Search terms** |
| --- | --- |
| Hand surgery search terms | (“hand/surgery”[mesh] OR ((surger∗[tiab] OR surgic∗[tiab]) AND (hand[tiab] OR hands[tiab] OR elbow[tiab] OR wrist∗[tiab] OR finger∗[tiab] OR thumb∗[tiab])) OR (“hand”[mesh] AND surgery[sh])) |
| SDOH search terms | (“place of residence” OR “insurance” OR “socioeconomic” OR “occupation” OR “employment status” OR “race” OR “ethnicity” OR “religion” OR “education” OR “social capital” OR “social determinants” OR “gender” OR “sex”) |
| Complete MEDLINE/PubMed query | ("hand/surgery"[MeSH Terms] OR (("surger"[Title/Abstract] OR "surgic"[Title/Abstract]) AND ("hand"[Title/Abstract] OR "hands"[Title/Abstract] OR "elbow"[Title/Abstract] OR "wrist"[Title/Abstract] OR "finger"[Title/Abstract] OR "thumb"[Title/Abstract])) OR ("hand"[MeSH Terms] AND "surgery"[MeSH Subheading])) AND ("place of residence"[All Fields] OR "insurance"[All Fields] OR "socioeconomic"[All Fields] OR "occupation"[All Fields] OR "employment status"[All Fields] OR "race"[All Fields] OR "ethnicity"[All Fields] OR "religion"[All Fields] OR "education"[All Fields] OR "social capital"[All Fields] OR "social determinants"[All Fields] OR "gender"[All Fields] OR "sex"[All Fields]) |
