# Supplementary material for: Quality Measures Addressing Disparities to Improve Outcomes in Hand Surgery
Source: Hand (N Y). 2026 Jun 23:15589447261453556. Online ahead of print. doi: 10.1177/15589447261453556 (PMC13294116; doi:10.1177/15589447261453556)
Supplement: sj-docx-2-han-10.1177_15589447261453556 – Supplemental material for Quality Measures Addressing Disparities to Improve Outcomes in Hand Surgery [file sj-docx-2-han-10.1177_15589447261453556.docx]

**Appendix Table A2:** Healthy People 2030 Social Determinants of Health Domains, Goals, and Related Objectives^1^

| **Healthy People 2030 SDOH Domain** | **Goal and Description** |
| --- | --- |
| Health Care Access and Quality | **Goal:** Increase access to comprehensive, high-quality health care services.  Healthy People 2030 aims to improve health by increasing timely, high-quality health care services. Examples of related objectives include reducing the proportion of emergency department visits with a longer than recommended wait time and increasing the proportion of adolescents who have had a preventative healthcare visit in the past year. |
| Social and Community Context | **Goal:** Increase social and community context.  Healthy People 2030 aims to improve support for people’s interpersonal relationships and interactions with other individuals. Examples of related objectives include increase the proportion of adolescents who have an adult they can talk to about serious problems and reduce anxiety and depression in family caregivers of people with disabilities. |
| Education Access and Quality | **Goal:** Increase educational opportunities and help children and adolescents do well in school.  Healthy People 2030 aims to provide high-quality educational opportunities for children and adolescents, as they note people with higher education levels are likely to be healthier and live longer. Examples of related objectives include increase the proportion of high school students who graduate in 4 years and increase the proportion of children who participate in high-quality and early childhood programs. |
| Economic Stability | **Goal:** Help people earn steady incomes that allow them to meet their health needs.  Healthy People 2030 aims to help more people achieve economic stability, as many people are unable to afford food, healthcare, and housing in the United States. Some examples of related objectives include reduce the proportion of people living in poverty, increase employment in working-age people, and reduce the proportion of families that spend more than 30% of their income on housing. |
| Neighborhood and Built Environment | **Goal:** Create neighborhoods and environments that promote health and safety.  Healthy People 2030 aims to improve neighborhoods that individuals live in given the effect on individual health. Some examples of related objectives include increasing the proportion of schools with policies and practices that promote health and safety and reduce the number of toxic pollutants released into the environment. |

*all information directly from Healthy People 2030.
